# Phosphorus Fertilization and Chemical Root Pruning: Effects on Root Traits During the Nursery Stage in Two Mediterranean Species from Central Chile

**DOI:** 10.3390/plants14020195

**Published:** 2025-01-12

**Authors:** Fiorella Calderón-Ureña, Carolina Álvarez-Maldini, Manuel Acevedo, Manuel E. Sánchez-Olate, R. Kasten Dumroese, Antay Sierra-Olea, Juan F. Ovalle, Edwin Esquivel-Segura

**Affiliations:** 1 Departamento de Silvicultura, Facultad de Ciencias Forestales, Universidad de Concepción, Concepción 4070374, Chile; caalvarez@udec.cl (C.Á.-M.); msanche@udec.cl (M.E.S.-O.); 2 Centro Tecnológico de la Planta Forestal, Instituto Forestal, Sede Biobío, San Pedro de la Paz 4130946, Chile; macevedo@infor.cl (M.A.); antaysierra.o@gmail.com (A.S.-O.); 3 Laboratorio de Cultivo de Tejidos Vegetales, Centro de Biotecnología, Universidad de Concepción, Concepción 4070374, Chile; 4 USDA Forest Service, Rocky Mountain Research Station, Moscow, ID 83843, USA; kas.usfs@gmail.com; 5 Laboratorio de Restauración de Bosques, Departamento de Silvicultura y Conservación de la Naturaleza, Universidad de Chile, Santiago 8820808, Chile; juan.ovalle@uchile.cl; 6 Center of Applied Ecology & Sustainability (CAPES), Santiago 8820808, Chile; 7 Escuela de Ingeniería en Agronomía, Campus Tecnológico Local San Carlos, Tecnológico de Costa Rica, Alajuela 22321001, Costa Rica; eesquivel@itcr.ac.cr

**Keywords:** fine roots, specific root length, root architecture, maqui, quillay

## Abstract

The role of a plant root system in resource acquisition is relevant to confront drought events caused by climate change. Accordingly, nursery practices like phosphorous (P) fertilization and root pruning have been shown to modify root architecture; however, their combined benefits require further investigation in Mediterranean species. We evaluated the effect of applied P concentrations (0, 15, 60, and 120 mg L^−1^ P) with or without chemical (copper) root pruning (WCu, WoCu, respectively) in *Aristotelia chilensis* and *Quillaja saponaria* on morpho-physiological and root architecture traits. Higher P concentration increased nutrient content in both species concurrent with higher growth. In *A. chilensis*, higher P concentrations only increased the length and volume of medium roots. In *Q. saponaria*, P additions increased root length and diameter and the length and volume of fine and medium roots. The root-to-shoot ratio declined with WCu in *A. chilensis* (23.1%) and *Q. saponaria* (15.7%). Unlike our hypothesis, fine root architecture remained unaffected with root pruning in *A. chilensis*, while fine root length and volume decreased with increasing P concentrations in *Q. saponaria*. Thus, P fertilization enhances root development more consistently than root pruning, highlighting the need for further testing under water deficit conditions to optimize nursery practices.

## 1. Introduction

Mediterranean regions are considered highly vulnerable to climate change [1,2]. The Mediterranean region of south–central Chile is considered a biodiversity hotspot [3] and is being negatively impacted by the combination of prolonged drought, increasing temperatures, and decreasing amounts of precipitation [1,4,5,6]. Such disturbances constitute a threat to biodiversity by reducing an ecosystem’s ability to acclimate to these new environmental conditions [7,8]. To improve the adaptation of ecosystems to climate change, especially forest ecosystems, forest restoration has been considered a key tool by contributing to a reduction in greenhouse gases and playing a role in mitigation [9]. However, the combination of inadequate seed supply [10], the low quantity and poor quality of available seedlings produced in nurseries, and low field survival [11,12] continues to limit the success of restoration in Mediterranean ecosystems [13].

In this regard, the Target Plant Concept seeks to define the adequate morpho-physiological plant attributes that favor increased survival and growth according to limiting environmental factors in the field [14,15,16,17,18,19,20,21]. Because of seasonal water scarcity in Mediterranean ecosystems, root architecture attributes are highly relevant, because, in addition to anchoring, the absorption of water and nutrients are fundamental for plant survival and growth after establishment [22]. For Mediterranean species, plant survival in drought events depends largely on the size of the root system and its ability to reach water stored in deeper soil horizons [23]. Nevertheless, initial root development in the field is mainly determined by the root architecture developed during the nursery stage [24,25,26] concurrently with plant nutrient concentrations [23]. Nursery practices that maximize root attributes have, however, been scarcely documented.

Phosphorus (P) is an essential nutrient for plant development, being a fundamental component in nucleic acids, membrane phospholipids, and energy-dependent metabolic processes [27]. Phosphorous has been traditionally described as a root growth stimulator [28], but few studies have documented such an effect. In fertilization, additions of high amounts of P to *Fraxinus mandshurica* led to an increased primary root length, while P deficit promoted lateral root development [29]. In shrub species, an increase in applied P yielded higher total root length, surface area, and root dry mass in *Bauhinia faberi* [30], and root specific length in *Pistacia lentiscus* [31]. Furthermore, in Mediterranean tree species such as *Quercus ilex*, P fertilization increased root biomass [32], which also correlated with field survival, as shown in a study with *Acacia salicina* [33].

Along with P fertilization, chemical root pruning is also related to changes in root architecture. This practice is based on the application of compounds, such as copper (Cu), on the interior surfaces of the container, where the meristem of lateral roots ceases growth when coming into contact with the wall, thus promoting a more fibrous root system due to the loss of apical dominance [14,34,35]. This treatment avoids root spiraling inside the container [14,36,37] and is linked to an increase in the number of lateral roots [38], a higher number of growth points [39], and a more fibrous root system [40]. Such attributes could favor plant survival [23] because root growth after establishment increases water and nutrient uptake and promotes photosynthesis that supports root and shoot growth [41]. Other studies have shown, however, that Cu did not avoid spiral root growth and decreased root length and surface area in roots smaller than 5 mm diameter, thus decreasing total root length and surface area [42].

Although P fertilization and chemical root pruning influence root architecture, the response of the combination of these nursery practices in woody, Mediterranean species, and their possible effect when facing water deficit, is scarce. Accordingly, our hypothesis is that seedlings exposed to chemical root pruning in concert with P fertilization will develop a more fibrous root system with longer roots in smaller diameter classes (i.e., <2 mm). Our aim is to evaluate the effect of applied P concentration and chemical root pruning in nursery-produced plants of *Aristotelia chilensis* and *Quillaja saponaria*, notably effects on morphological attributes, nutrient status, and root architecture. A pioneer native shrub from south–central Chile, *A. chilensis*, is desired for restoration purposes because its seeds germinate readily and abundantly [43], inducing high plant density when established in areas devoid of vegetation [44]. A Chilean endemic, *Q. saponaria* is one of the most produced native species in nurseries because its bark is a commercial source of saponin [45,46]. Found mainly in warm and dry climates, it is one of the most abundant species in the sclerophyllous forest of Mediterranean central Chile, because it also occupies cold and wet sites [47]. Despite their ecological and commercial importance, nursery production of these species has not focused on maximizing their root architecture to address water deficit after outplanting.

## 2. Results

### 2.1. Morphological Attributes

At the end of the nursery stage, P concentration was significant for root and leaf biomass, leaf area, and R:S (Table 1). The control (0P) had the lowest leaf area compared to all other P concentrations; the same trend was observed for leaf biomass. The highest P concentration (120P) yielded more root biomass than 0P (Table 2). In contrast, root pruning was significant only for root biomass and R:S; the control treatment (WoCu) yielded more root biomass than WCu, and the same trend was observed for R:S (Table 2). The interaction of P concentration and root pruning was significant for *A. chilensis* stem biomass (Table 1); for 0P, stem biomass was the same regardless of root pruning treatment, but at 120P, the WCu treatment had more stem biomass than the WoCu treatment (Table 2).

For *Q. saponaria*, P concentration was significant for all morphological traits except R:S (Table 1). In general, the highest concentrations of P (120 and 60) had higher amounts of leaf area and leaf, stem, and root biomass than 60P, which had greater values for these variables than 0P. Root pruning was significant for leaf area, leaf and stem biomass, and R:S; leaf area, and leaf and stem biomass were higher with copper pruning (WCu) but R:S was lower compared to WoCu (Table 2). No interaction of P concentration and root pruning was observed.

**Figure 1:**
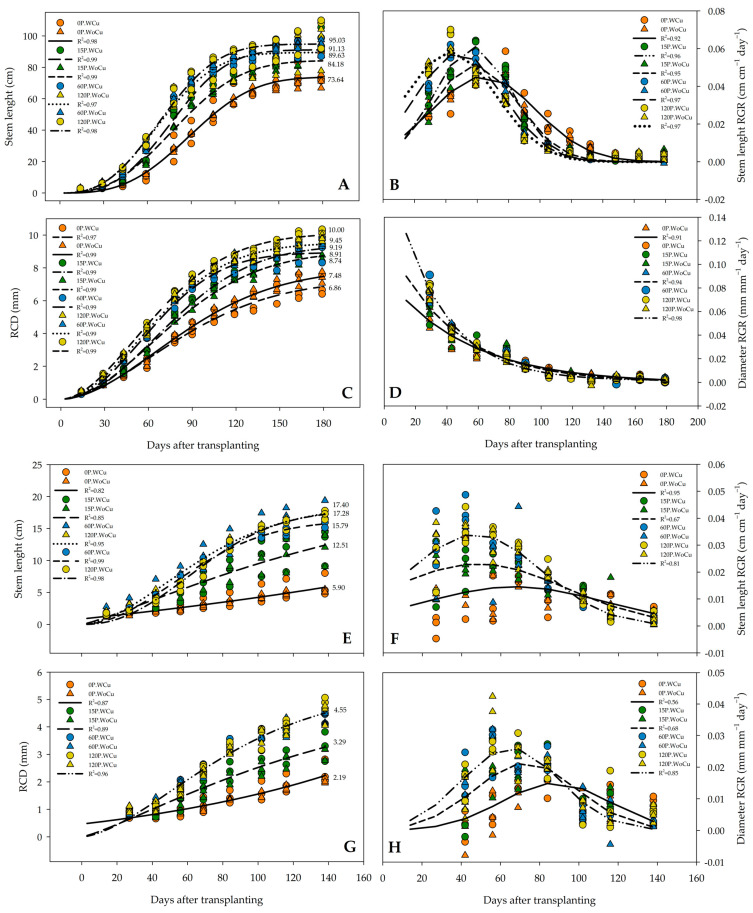
Growth dynamics of stem length and root collar diameter (RCD) for *Aristotelia chilensis* (**A**,**C**) and *Quillaja saponaria* (**E**,**G**), in relation to applied phosphorous concentration (0, 15, 60, and 120 mg L^−1^ P), chemical root pruning (with, WCu, and without, WoCu), and their interaction (n = 24 for each treatment). Figures on the right illustrate the relative growth rate (RGR) of stem height and RCD for *A. chilensis* (**B**,**D**) and *Q. saponaria* (**F**,**H**). Colored symbols indicate the mean, and lines indicate modeled response for treatments.

In *A. chilensis*, the RGR of the 120P concentration revealed that maximum growth occurred sooner (43 days after transplanting) than that for 60P, 15P, and 0P (45, 59, and 61 days, respectively) (Figure 1B). The 0P-WoCu and the 0P-WCu treatments promoted the least amount of stem length during the entire growth season compared to all the other treatment combinations (Figure 1A). For RCD growth, only the 60P-WoCU and 120P-WoCu treatments shared similar growth rates throughout the nursery stage (Figure 1C). Whereas the rate of stem length growth increased during the nursery stage, the rate of RCD was greatest early in the nursery stage and then declined (Figure 1D), regardless of treatment.

In *Q. saponaria*, the 120P, 60P, and 15P concentrations (regardless of root pruning) reached maximum stem length RGR at day 50, 20 days sooner than the control (0P) (Figure 1F). The RCD reflected effects on P concentrations but not root pruning treatment (Figure 1G). The highest P concentrations (120 and 60) generated the most RCD throughout the growing period, and these rates achieved their maximum growth rate 69 days after transplanting, faster than the 71 days for 15P and 87 days for 0P (Figure 1H). Also, 0P and 15P concentrations, irrespective of root pruning treatment, and the 60P-WoCu and 120P-WoCu treatments shared the highest stem length dynamic (Figure 1E). The 120P-WoCu and 60P-WoCu treatments showed the maximum stem length, while the lowest stem length was observed in the 0P-WoCu and 0P-WCu treatments.

### 2.2. Nutrient Concentration and Content Analysis

In *A. chilensis*, the level of applied P concentration was significant for plant N, P, and K concentrations and N and P content (Table 1). The control (0P) had the lowest N concentration and content compared to all other applied P concentration treatments. Not surprisingly, sequential increases in applied P concentration increased plant P concentration and content. In contrast, sequential increases in applied P concentrations decreased K concentrations (Table 3). The root pruning treatment was significant for plant N and P concentrations (Table 1). A higher N concentration was observed in the WCu treatment compared to the WoCu treatment; the opposite behavior was observed for plant P concentration (Table 3). Also, no significant interaction between applied P concentration and root pruning was observed for plant nutrient concentration and content.

For *Q. saponaria*, applied P concentration was significant for K concentration, and N, P, and K contents (Table 1). Potassium foliar concentration was highest in the 15P concentration treatment and lowest in the 0P control treatment, independent of root pruning treatment. As a general trend, N, P, and K contents were higher in the 120P concentration and lowest in the 0P (Table 3). Root pruning was not significant for nutrient concentrations or contents. The applied P concentration × root pruning treatment interaction was significant for plant N and P concentrations (Table 1). The highest N concentrations were observed in the 0P-WoCu treatment followed by the 0P-WCu treatment, while the 60P-WoCu, 60P-WCu, and 120P-WCu treatments displayed the lowest N concentrations (Table 3). Regarding plant P concentration, higher values were observed in 15P-WoCu, while the lowest values were found in the 15P-WCu treatment (Table 3). As a general trend, the presence of copper pruning resulted in lower plant P concentrations than that in seedlings grown without copper pruning.

### 2.3. Root Architecture Traits

For *A. chilensis*, P concentration was not significant for any of the root architecture traits (Table 1), whereas root pruning was significant for root volume. The WoCu treatment increased root volume by 33% (10.15 cm^3^) compared to the WCu treatment (7.83 cm^3^).

**Figure 2:**
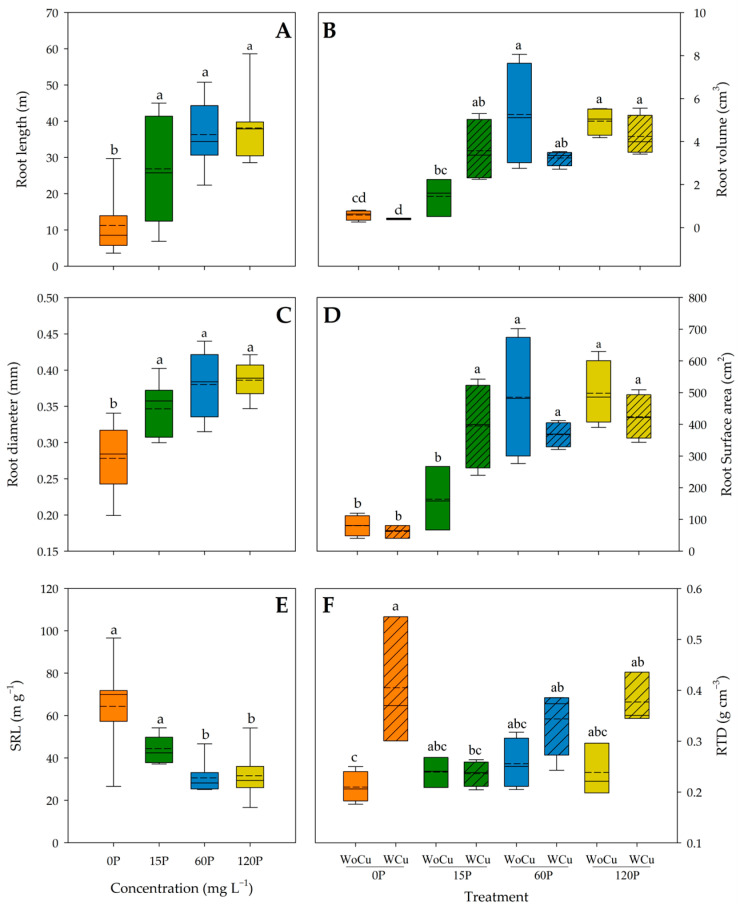
Root length (**A**), volume (**B**), diameter (**C**), surface area (**D**), specific root length (SRL) (**E**), and root tissue density (RTD) (**F**) of *Quillaja saponaria* in relation to applied phosphorous concentration (0, 15, 60, and 120 mg L^−1^ P), chemical root pruning (without, WoCu, and with, WCu), and their interaction (n= 8 for each treatment). The bottom and top boundaries of the boxes represent the 25th and 75th percentiles, respectively. The solid dashed on the center of each box represents the mean value and the solid line represents the median value. Different letters indicate significant differences among treatments at *p* < 0.05 according to Tukey.

For *Q. saponaria*, the P concentration was significant for root length, root diameter, and SRL (Table 1). Thus, root length and diameter were higher in any treatments with *p* > 0 compared to the control (0P) (Figure 2A and Figure 2C, respectively).

On the contrary, the 60P and 120P concentrations had a greater SRL than their 0P and 15P counterparts (Figure 2E). Root pruning only affected the SRL (Table 1), with higher values in the WoCu treatment (47.22 ± 23.80 m g^−1^) compared to WCu (36.66 ± 15.52 m g^−1^). The interaction of P concentration and root pruning treatments was significant for root volume, root surface area, and RTD (Table 1). The lowest root volume was observed in the 0P concentration, followed by the 15P-WoCu treatment, while the 120P concentration showed the highest root volume (Figure 2B). Similarly, the 0P and the 15P-WoCu treatments had the lowest root surface area, compared to all the other treatments (Figure 2D). For RTD, the 0P-WoCu and 15P-WCu treatments had significantly lower tissue density, while the highest RTD was observed in the 0P-WCu treatment (Figure 2F).

**Figure 3:**
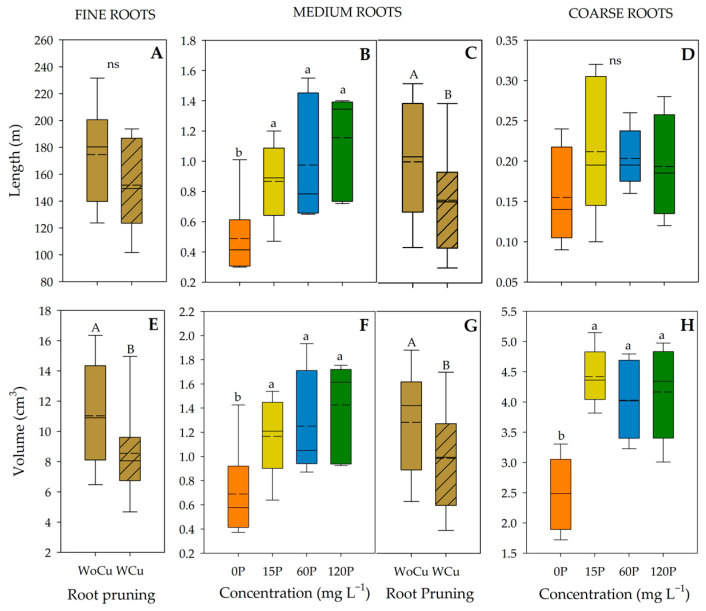
Root length (**A**–**D**) and volume (**E**–**H**) of *Aristotelia chilensis* in relation to applied phosphorous concentration (0, 15, 60, and 120 mg L^−1^ P) and chemical root pruning (without, WoCu, and with, WCu) according to the diameter distribution (fine, medium, and coarse roots) (n = 4 for each treatment). The bottom and top boundaries of the boxes represent the 25th and 75th percentiles, respectively. The dashed line in the center of each box represents the mean value and the solid line represents the median value. Different letters indicate significant differences between treatments at *p* ≤ 0.05 according to Tukey; ns = non-significant.

For *A. chilensis* root traits by size class, P concentration was significant for the length of medium-sized roots, and the volume of medium- and coarse-sized roots (Table 4). When P was applied (i.e., 120P, 60P, or 15P), medium-sized roots were longer than those in the control (Figure 3B). The same trend was observed for the volume of medium- and coarse-sized roots (Figure 3F and 3H, respectively). Root pruning was significant for the length of medium-sized roots, and the volume of fine- and medium-sized roots (Table 4), where shorter lengths and lower volumes were observed in the WCu treatment (Figure 3C, 3E, and 3G, respectively). Also, no interaction between P concentration and root pruning was observed.

**Figure 4:**
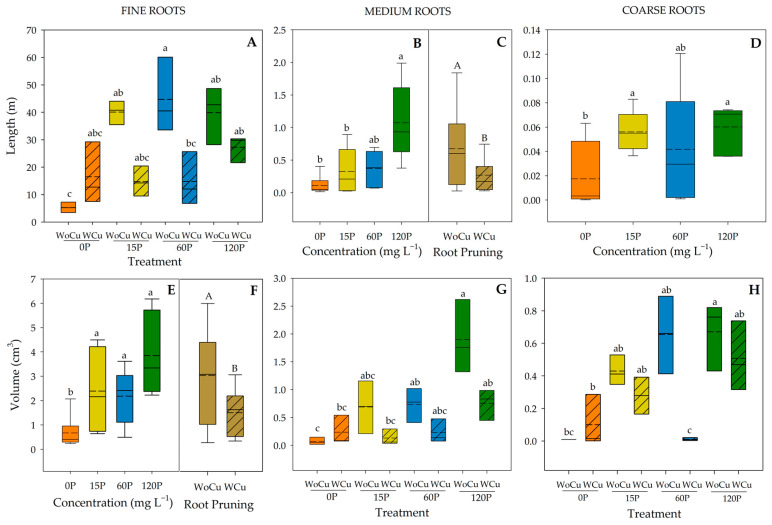
Root length (**A**–**D**) and volume (**E**–**H**) of *Quillaja saponaria* in relation to applied phosphorous concentration (0, 15, 60, and 120 mg L^−1^ P), chemical root pruning (without, WCu and without, WoCu), and their interaction according to the diameter distribution (fine, medium, and coarse roots) (n = 8 for each treatment). The bottom and top boundaries of the boxes represent the 25th and 75th percentiles, respectively. The dashed line in the center of each box represents the mean value and the solid line represents the median value. Different letters indicate significant differences between treatments at *p* ≤ 0.05 according to Tukey.

For *Q. saponaria*, P concentration was significant for the length of medium- and coarse-size roots, and the volume of fine-size roots (Table 4). The 120P concentration showed a greater length compared to 15P and 0P in medium-size roots (Figure 4B). The 120P and 15P concentrations had the greatest length in coarse-size roots; meanwhile, the control (0P) showed the lowest (Figure 4D). All P concentrations (120P, 60P, or 15P) had a higher root volume than the 0P concentration (Figure 4E). Root pruning was significant for the length of medium-sized roots and volume of fine-sized roots (Table 4), where shorter lengths and lower volumes were observed in the WCu treatment (Figure 4C and 4F, respectively). The interaction of P concentration and root pruning was significant for the length of fine-sized roots and volumes of medium- and coarse-sized roots (Table 4). The lowest length in fine-size roots was observed in the 0P-WoCu treatment and the highest in 60P-WoCu (Figure 4A). For medium-sized roots, 0P-WoCu treatment yielded a significantly lower volume, while the highest was observed in 120P-WoCu (Figure 4G). Similarly, the 120P-WoCu treatment had the highest volume in the coarse-size roots and 60P-WCu showed the lowest (Figure 4H).

## 3. Discussion

In *A. chilensis*, we observed that increasing P concentration applied during nursery production significantly increased RGR, resulting in greater plant growth and size. The increase in growth is well correlated with the increase in N and P content observed in the applied P concentrations, and this effect has been previously observed in *Acer mono* and *Quercus ilex* [48,49]. In our study, applied P concentration increased leaf area (47%) and leaf biomass (51%) more than root biomass (21%) relative to the 0P control treatment, thus causing a decrease in the R:S. Similar results were observed in *Araucaria angustifolia* cultivated with concentrations greater than 118 mg L^−1^ P [50]. The decrease in the R:S could induce an imbalance between water absorption and transpiration, leading to water stress and decreased survival after field establishment [51,52]. Despite that, increasing P concentration also promoted higher root biomass, which agrees with other research [30,32,33]. This was, however, not related to changes in the architecture of fine roots in *A. chilensis*, which are mainly responsible for water and nutrient absorption [30,53,54,55]. Contrary to our expectations, the increase in medium- and coarse-sized root length and volume observed with increases in applied P concentration were mostly responsible for the observed increase in total root biomass.

The chemical (i.e., copper) root pruning decreased *A. chilensis* root biomass, which agrees with several reports [40,42,56]. The objective of chemical root pruning is to limit the growth of lateral roots and stimulate the growth of higher-order roots [34,35], thus developing a fibrous root system with higher growth of shorter and thinner roots [40,42]. Some studies have shown no differences in root architecture [14,38]. However, contrary to our hypothesis, root pruning did not increase the volume or length of fine roots as expected, and significantly decreased the length and volume of medium roots, thus explaining the overall lower root biomass in this treatment. This also led to a decline in R:S, which, as previously discussed, could negatively impact plant performance in drought stress conditions.

Similarly to the results observed in *A. chilensis*, increasing the rate of applied P to *Q. saponaria* increased stem length, diameter, biomass, and leaf area. Contrary to *A. chilensis*, P concentration had, however, no effect on the R:S. Higher applied P concentration also enhanced plant N, P, and K content, which is associated with increased growth and above- and below-ground biomass. Similar results were observed in *Eucalyptus grandis*, where plants exhibited higher shoot growth and promoted accumulation of N, P, and K content with increased applied P concentration [57]. The development of more leaf area and shoot biomass with increasing applied P has also been reported in *Swietenia macrophylla* [58]. Although the effects of P have been commonly researched by assessing the effects of its deficiency, contrasting results have been observed in leaf area, shoot biomass, and R:S in different species [31,49,59,60], indicating that the effects of P on plant growth and biomass distribution are highly species-specific. This agrees with our distinct results observed between *A. chilensis* and *Q. saponaria*.

Although the applied P concentration interacted with root pruning, we noted that N foliar concentrations decreased as the rate of applied P increased. Similar results have been recorded for *E. grandis*; the effect was described as antagonism between phosphate and nitrate (NO_3_^−^ [61]. Similar results were observed in *Phoebe zhennan* [62]. However, lower N concentrations caused by an increased rate of applied P in our study had no implications on growth and biomass accumulation, probably due to higher N content in P-fertilized plants.

Contrary to our results for *A. chilensis*, P concentration and chemical root pruning had major effects on *Q. saponaria* root architecture. The literature shows that P deficiency induces a decrease in root diameter and an increase in root length toward expanding the area of soil explored, thus increasing root surface area (RSA) [63,64,65,66,67] in trees and shrubs [29,31]. However, in *Q. saponaria,* we observed that P deficiency in the 0P concentration decreased root diameter and length, in contrast to previous studies. The lower root diameter and root biomass led to an increase in SRL in the 0P control, which is commonly observed in P-deficient soils linked to higher P uptake efficiency [68,69] and increasing soil exploration volume per unit of carbon invested in root length [70,71,72,73]. Besides root diameter, a meta-analysis revealed that higher SRL correlated with decreased RTD [74]. In our study, however, this relationship depended on the application of chemical root pruning and the level of applied P. In our 0P concentration, the expected low RTD in concert with high SRL was noted in the WoCu treatment, but in WCu, RTD and SRL were both high. Although there was no difference in root diameter under P-deficient conditions, the application of Cu may promote the development of denser roots that might increase plant tolerance to drought conditions [74] and nutrient acquisition in poorly nourished soils after outplanting [71]. This combination of high SRL and RTD related to chemical root pruning was absent when P was applied (i.e., 15P, 60P, and 120P). Additionally, the application of Cu induced a decrease in the length and volume of fine roots and showed a tendency to decrease the volume of medium and coarse roots, which can negatively affect nutrient and water absorption in resource-limited conditions.

The application of P induced a lower SRL in *Q. saponaria*, which agrees with results from several species [48,66,75]. Because a higher SRL has been linked to resistance to water stress [74,76,77] by increasing the efficiency in resource acquisition, these results could imply that P could induce lower drought resistance after establishment. However, in *Q. saponaria*, the lower SRL of P-fertilized plants was also linked to higher RSA, root diameter, root length, volume, RTD, and biomass, traits that are usually present in species subjected to water deficit or present in habitats with lower rainfall [59,78]. All these traits should confer high resource acquisition due to increased root–soil interface. The high nutrient content induced by P fertilization in *Q. saponaria* could imply a high resource investment in denser roots with longer lifespans, as described in roots with higher RTD [71,76,79,80]. Phosphorous fertilization also had a high impact on root architecture at different diameter distributions. More importantly, increasing P concentration induced the development of longer fine roots with increased volume, implying a larger volume of soil exploited per unit of biomass invested [74]. Fine roots play a significant role in soil exploration for water and nutrient acquisition and can account for up to 80% of the total root length [81,82]. Thus, minute changes in fine root architecture could result in great changes in plant performance after field establishment. Similarly, not accounting for the effect of chemical root pruning, higher P concentrations also increased the volume and length of medium- and coarse-sized roots. This could also have a beneficial effect in resource-limited conditions, because, beyond anchorage, coarse roots serve as a carbohydrate reserve and absorb water from deeper soil horizons [81,83].

Although *A. chilensis* and *Q. saponaria* had effects due to P concentrations and RP, the rapid shoot and root growth of *A. chilensis* was unexpected. This growth response resulted in the roots of this species densely occupying the entire container substrate by the end of the nursery period (Figure S1), and that might have limited the accurate root development response for the effects of P and RP. Meanwhile, for *Q. saponaria* at the conclusion of the nursery phase, root plugs were intact, but roots were not densely packed, allowing for the determination of differences in response to applied treatments (Figure S2).

## 4. Materials and Methods

### 4.1. Seedling Establishment in the Nursery

We grew seedlings of *A. chilensis* and *Q. saponaria* in an outdoor nursery at the Centro Tecnológico de la Planta Forestal, Instituto Forestal (36.84° S; 73.13° W), Region of Biobío, Chile. During the nursery period (November 2022–May 2023), the mean daily temperature was 16.8 °C. The maximum daily temperature (34.4 °C) was registered in February and the minimum (0 °C) in May. Seeds of both species were collected in El Morro (38.03° S; 72.68° W) and Quillón (36.74°S; 72.49° W), Region of Biobío, respectively. In late August 2022, seeds were soaked in water for 48 h, sown into germination beds filled with composted pine bark [84,85], and irrigated daily (Table S1). When seedlings developed their first set of true leaves (*A. chilensis*, end of October 2022; *Q. saponaria*, beginning of January 2023), we transplanted them individually into 32 trays per species (15 cm depth, 280 cm^3^ volume, 24 cavities; 768 seedlings) that had either been treated with a solution of 60 g L^−1^ Cu_2_(OH)_3_Cl applied to the internal wall of the cavities (16 trays) for chemical root pruning treatment (WCu) or not (16 trays, WoCu). The trays were filled with composted pine bark as described above.

### 4.2. Fertilization

Phosphorous treatments began one month after transplanting. We applied 4 P concentrations: 0, 15, 60, and 120 mg L^−1^, hereafter 0P, 15P, 60P, and 120P. Macro- and micronutrients were applied at constant concentrations (mg L^−1^): nitrogen (N, 400), potassium (K, 100), magnesium (Mg, 60), sulfur (S, 80), calcium (Ca, 80), iron (Fe, 10), manganese (Mn, 6), copper (Cu, 2), zinc (Zn, 6), molybdenum (Mo, 2), and boron (B, 2). Ammonium nitrate was the N source (proportion 1NO_3_^−^ to 1NH_4_^+^). Available water was estimated using soil humidity sensors (ECH20 EC-5; Decagon, Pullman, WA, USA); sensor values for volumetric water content (m^3^ m^−3^) were calibrated with the gravimetric mass to estimate the percentage of available water [86]. We applied phosphorous and the other nutrients as soluble fertilizer (fertigation) and alternated with irrigation events each time trays reached 50% of available water. Fertigation and irrigation were manually applied with a watering can until full container capacity was reached. We added macronutrients to every fertigation and micronutrients during every third fertigation. The N concentration of *A. chilensis* was reduced to 200 mg L^−1^ 96 days after transplanting.

Our experimental design for each species was a completely randomized factorial design: 4 P concentrations (0P, 15P, 60P, and 120P) × 2 chemical root pruning treatments (WoCu and WCu) × 4 replicates (trays) × 24 seedlings tray^−1^ = 768 seedlings total.

### 4.3. Morpho-Physiological Evaluations

Starting on day 14 after transplanting and continuing about every 2 weeks to the end of the nursery phase (12 and 9 measurements for *A. chilensis* and *Q. saponaria*, respectively), we selected 192 seedlings of each species (8 seedlings × 4 P concentrations × 2 root pruning × 3 replicates) to determine stem length (SL, cm) and root collar diameter (RCD, mm). The measurements of SL and RCD were used to calculate the relative growth rate [87,88].

At the end of the nursery stage (June 2023; *A. chilensis*, 180 days; *Q. saponaria*, 140 days after transplanting), 64 seedlings of each species were randomly selected (2 seedlings × 4 P concentrations × 2 root pruning × 4 replicates) for root and shoot morphological assessment. Roots were separated from shoots, gently washed, dyed with a crystal violet solution (5 g L^−1^), quantified with a high-resolution scanner (1200 DPI resolution, Epson Perfection 4490 Scanner^®^, Nagano, Japan), and analyzed with a root image analysis system (WinRhizo^®^, Regent Instrument Inc., Québec City, QC, Canada). The means for total root diameter, length, surface area, and volume were determined, as well as the distribution of roots among diameter classes (fine roots ˂ 1 mm, medium roots = 1–2 mm, and coarse roots ˃ 2 mm) [24]. The area of fresh leaves (cm^2^) was measured using a leaf area meter (LI-3100, LI-COR Biosciences, Lincoln, NE, USA). We dried shoots (i.e., stems and leaves) and roots at 60 °C for 72 h in a forced-ventilation oven to determine the dry mass per component. The root-to-shoot ratio (R:S) was calculated as the root dry mass × shoot dry mass^−1^. We calculated specific root length (SRL, m g^−1^) as root length × root dry mass^−1^, and the root tissue density (RTD, g cm^−3^) as root dry mass × root volume^−1^. The specific leaf area (SLA, cm^2^ g^−1^) was calculated as the foliar area × leaf dry mass^−1^. The same suite of measurements with the same number of seedlings was used for *A. chilensis*, except that only 32 seedlings were used for root architecture (1 seedling × 4 P concentrations × 2 root pruning × 4 replicates).

We selected 3 foliar samples per treatment per species (4 P concentrations × 2 root pruning; 24 total) to obtain N, P, and K concentrations per species. We used the Kjeldahl digestion method and colorimetry for N [85]. For P and K, we prepared samples using dry calcination, and P concentration was determined using colorimetric dissolution in 1 N hydrochloric, while K was determined by atomic emission spectrophotometry [85]. Nutrient concentrations and leaf dry mass were used to obtain nutrient content.

### 4.4. Data Analysis

The SL, RCD, and RGR data during the nursery stage (180 days for *A. chilensis* and 140 for *Q. saponaria*) were modeled with the PROC NLIN procedure (SAS Institute Inc., Cary, NC, USA) using the Gauss–Newton method through a derivative-free algorithm. For SL and RCD, we adjusted a Weibull model for all treatments, with the exception of the SL in the 0P concentration of *Q. saponaria*, where an Asymptotic Regression model was adjusted. For RGR, we used a Gauss Peak Shape model for all treatments, except for RCD in *A. chilensis*, where a First-order Decay Kinetics model was adjusted.

The effect of P concentration and root pruning at the model level was evaluated using the additional sums of squares method [89]. The final morphological attributes, leaf area, SLA, root architecture (i.e., root diameter, length, volume, and surface area), SRL, RTD, biomass (leaves, stem, roots, and R:S), and nutrient concentration and content were assessed using a two-way analysis of variance (ANOVA) for a completely randomized design, through PROC GLIMMIX (SAS Institute Inc., Cary, NC, USA) with a distribution selected considering the Akaike Information Criterion (AIC). Statistical differences between means were performed with the Tukey test (HSD) for multiple comparisons with 95% confidence. All visualizations were made with graphing software (SigmaPlot 14.0, Systat Software Inc., San José, CA, USA).

## 5. Conclusions

Our study shows that the efficacy of nursery treatments, namely applied P concentration and use of chemical (i.e., copper) root pruning, both of which aim to modify root architecture traits, is species-specific. We observed that *A. chilensis* fully colonized the container substrate during nursery production, which could have influenced the lack of response in root pruning treatments. This shows the need to account for the growth dynamics of Mediterranean species to select suitable containers and/or fertility regimes for optimal development in the nursery. Contrary to our hypothesis, P concentration did not affect the development of fine roots and only had minor effects on the length and volume of medium roots. Regarding *Q. saponaria* and in agreement with our hypothesis, P concentration induced the expected changes in root architecture, especially in fine- and medium-sized roots. Considering whole root architecture, P concentration induced changes that could relate to higher plant performance under water-limited conditions and led to an improvement in plant nutritional status, which has been extensively correlated to better plant performance in Mediterranean climates. However, the implications of these changes on plant performance require further evaluation. In disagreement with our hypothesis, chemical root pruning prompted changes that could negatively impact plant performance, mostly in fine- and medium-sized roots. Considering that root pruning during nursery production is costly and time-consuming, our results do not present a clear benefit of its application toward improving root systems to increase drought avoidance.

## Figures and Tables

**Table 1:** *p*-values of morphological attributes, nutrient concentrations and content, and root architecture in *Aristotelia chilensis* and *Quillaja saponaria* in relation to applied phosphorous concentration (P), chemical root pruning (RP), and their interaction. R:S: root-to-shoot ratio; SRL: specific root length; RTD: root tissue density. Significant values at *p* < 0.05 in bold.

	Variable	*Aristotelia chilensis*			*Quillaja saponaria*		
		Phosphorous (P)	Root Pruning (RP)	P × RP	Phosphorous (P)	Root Pruning (RP)	P × RP
Morphological	Leaf area	**˂** **0.0001**	0.2788	0.9136	**˂** **0.0001**	**0.0057**	0.4347
	Leaf biomass	**˂** **0.0001**	0.1892	0.8667	**˂** **0.0001**	**0.0029**	0.3596
	Stem biomass	˂0.0001	0.0045	**0.0006**	**˂** **0.0001**	**0.0004**	0.4996
	Root biomass	**0.0404**	**0.0086**	0.3849	**˂** **0.0001**	0.1606	0.5951
	R:S	**˂** **0.0001**	**0.0041**	0.8804	0.0522	**0.0228**	0.8139
Nutritional	N concentration	**˂** **0.0001**	**0.0006**	0.1396	˂0.0001	0.0001	**0.0131**
	P concentration	**˂** **0.0001**	**0.0124**	0.1630	0.0016	0.0012	**0.0074**
	K concentration	**˂** **0.0001**	0.1950	0.8049	**0.0043**	0.9467	0.1298
	N content	**˂** **0.0001**	0.1172	0.8296	**˂** **0.0001**	0.3275	0.8368
	P content	**˂** **0.0001**	0.5542	0.9462	**˂** **0.0001**	0.2370	0.7441
	K content	0.0552	0.8510	0.7362	**˂** **0.0001**	**0.0249**	0.4022
Root architecture	Root length	0.2742	0.2417	0.4801	**˂** **0.0001**	0.1532	0.1353
	Root diameter	0.1095	0.1468	0.5625	**˂** **0.0001**	0.2709	0.3179
	Root volume	0.0785	**0.0364**	0.4313	˂0.0001	0.8007	**0.0099**
	Root surface area	0.1601	0.0616	0.4043	˂0.0001	0.4938	**0.0140**
	SRL	0.4362	0.1305	0.5325	**0.0002**	**0.0350**	0.0862
	RTD	0.1140	0.8988	0.3191	0.1142	0.0001	**0.0263**

**Table 2:** Mean values (±standard deviation; n = 8) of leaf area (cm^2^) and leaf, stem, and root biomass (g) in *Aristotelia chilensis* and *Quillaja saponaria* in relation to applied phosphorous concentration (0, 15, 60, and 120 mg L^−1^ P), chemical root pruning (with, WCu, and without, WoCu), and their interaction. Different letters refer to significant differences between treatments at *p* ≤ 0.05 according to Tukey; ns = non-significant.

			Biomass (g)				
		Leaf Area (cm^2^)	Leaf	Stem		Root	R:S
				WoCu	WCu		
*A. chilensis*	Phosphorous (P)						
	0P	383.15 ± 102.21 ^b^	4.03 ± 0.45 ^b^	5.63 ± 0.61 ^d^	5.05 ± 0.53 ^d^	3.48 ± 1.87 ^b^	0.36 ± 0.08 ^a^
	15P	759.53 ± 217.65 ^a^	7.66 ± 1.52 ^a^	12.3 ± 0.10 ^c^	16.00 ± 2.62 ^ab^	4.28 ± 2.27 ^ab^	0.20 ± 0.05 ^b^
	60P	781.03 ± 218.31 ^a^	7.75 ± 1.28 ^a^	14.62 ± 1.28 ^abc^	14.98 ± 2.53 ^abc^	3.85 ± 2.05 ^ab^	0.18 ± 0.03 ^b^
	120P	803.55 ± 157.65 ^a^	8.26 ± 1.15 ^a^	14.35 ± 2.39 ^bc^	18.08 ± 1.81 ^a^	4.40 ± 2.32 ^a^	0.19 ± 0.04 ^b^
	Root Pruning (RP)						
	WoCu	667.37 ± 237.28 ^ns^	7.07 ± 1.98 ^ns^	11.72 ± 4.04 ^ns^		4.33 ± 2.25 ^a^	0.26 ± 0.10 ^a^
	WCu	700.79 ± 260.75 ^ns^	6.78 ± 2.13 ^ns^	13.53 ± 5.46 ^ns^		3.67 ± 1.95 ^b^	0.20 ± 0.08 ^b^
*Q. saponaria*	Phosphorous (P)						
	0P	13.37 ± 6.17 ^c^	0.21 ± 0.09 ^c^	0.06 ± 0.05 ^c^		0.14 ± 0.08 ^c^	0.65 ± 0.21 ^ns^
	15P	44.55 ± 19.47 ^b^	0.72 ± 0.36 ^b^	0.25 ± 0.15 ^b^		0.69 ± 0.44 ^b^	0.75 ± 0.15 ^ns^
	60P	87.95 ± 18.73 ^a^	1.49 ± 0.42 ^a^	0.52 ± 0.13 ^a^		1.21 ± 0.68 ^a^	0.61 ± 0.07 ^ns^
	120P	109.69 ± 44.19 ^a^	1.81 ± 0.92 ^a^	0.71 ± 0.43 ^a^		1.31 ± 0.76 ^a^	0.56 ± 0.07 ^ns^
	Root Pruning (RP)						
	WoCu	55.84 ± 39.26 ^b^	0.88 ± 0.66 ^b^	0.30 ± 0.23 ^b^		0.77 ± 0.56 ^ns^	0.70 ± 0.14 ^a^
	WCu	71.94 ± 50.34 ^a^	1.23 ± 0.94 ^a^	0.47 ± 0.41 ^a^		0.90 ± 0.63 ^ns^	0.59 ± 0.14 ^b^

**Table 3:** Mean values (±standard deviations; n = 3 for each treatment) of nitrogen (N), phosphorous (P), and potassium (K) nutritional concentration and content in *Aristotelia chilensis* and *Quillaja saponaria* in relation to applied phosphorous concentration (0, 15, 60, and 120 mg L^−1^ P), chemical root pruning (with, WCu, and without, WoCu), and their interaction. Different letters refer to significant differences among treatments at *p* ≤ 0.05 according to Tukey; ns = non-significant.

	Nutrient Concentration (mg g^−1^)						Nutrient Content (mg)		
*A. chilensis*	N		P			K	N	P	K
Phosphorous (P)									
0P	12.15 ± 1.76 ^b^		0.67 ± 0.12 ^d^			12.10 ± 2.30 ^a^	48.17 ± 4.49 ^b^	2.63 ± 0.22 ^c^	47.69 ± 4.34 ^ns^
15P	16.47 ± 2.76 ^a^		1.28 ± 0.15 ^c^			8.28 ± 0.90 ^b^	125.22 ± 23.96 ^a^	9.81 ± 1.80 ^b^	63.77 ± 14.95 ^ns^
60P	16.58 ± 1.01 ^a^		1.77 ± 0.12 ^b^			6.60 ± 0.50 ^c^	133.75 ± 17.85 ^a^	14.29 ± 2.39 ^a^	53.29 ± 7.81 ^ns^
120P	17.60 ± 1.87 ^a^		2.08 ± 0.23 ^a^			7.43 ± 0.80 ^bc^	136.97 ± 14.32 ^a^	16.52 ± 2.09 ^a^	58.89 ± 6.43 ^ns^
Root pruning (RP)									
WoCu	14.47 ± 2.65 ^b^		1.51 ± 0.57 ^a^			8.24 ± 1.97 ^ns^	105.56 ± 39.07 ^ns^	10.62 ± 5.66 ^ns^	55.87 ± 12.91 ^ns^
WCu	16.78 ± 2.44 ^a^		1.39 ± 0.58 ^b^			8.97 ± 2.95 ^ns^	116.50 ± 42.79 ^ns^	11.01 ± 5.95 ^ns^	55.95 ± 8.31 ^ns^
*Q. saponaria*	WoCu	WCu	WoCu		WCu				
Phosphorous (P)									
0P	34.43 ± 4.89 ^a^	27.33 ± 1.99 ^b^		2.10 ± 0.26 ^b^	2.06 ± 0.21 ^ab^	9.38 ± 1.21 ^b^	7.09 ± 1.82 ^c^	0.49 ± 0.16 ^c^	2.23 ± 0.82 ^c^
15P	20.67 ± 2.57 ^c^	18.27 ± 0.42 ^cd^		1.83 ± 0.25 ^a^	1.60 ± 0.17 ^c^	12.20 ± 1.25 ^a^	15.94 ± 3.88 ^b^	1.41 ± 0.40 ^b^	10.06 ± 2.61 ^b^
60P	15.90 ± 0.69 ^d^	16.20 ± 0.70 ^d^		1.80 ± 0.17 ^ab^	1.77 ± 0.06 ^bc^	10.75 ± 1.19 ^ab^	24.64 ± 3.92 ^a^	2.73 ± 0.35 ^a^	9.38 ± 1.81 ^b^
120P	21.07 ± 0.70 ^c^	15.47 ± 0.49 ^d^		2.43 ± 0.15 ^ab^	1.70 ± 0.17 ^bc^	11.12 ± 1.04 ^ab^	34.15 ± 8.84 ^a^	3.80 ± 0.76 ^a^	12.20 ± 6.83 ^a^
Root pruning (RP)									
WoCu	23.02 ± 7.59 ^ns^		2.04 ± 0.32 ^ns^			10.93 ± 1.87 ^ns^	19.13 ± 9.78 ^ns^	2.00 ± 1.28 ^ns^	10.97 ± 6.50 ^b^
WCu	19.32 ± 5.04 ^ns^		1.78 ± 0.23 ^ns^			10.79 ± 1.09 ^ns^	21.78 ± 13.10 ^ns^	2.22 ± 1.48 ^ns^	13.97 ± 9.45 ^a^

**Table 4:** *p*-values of root architecture in *Aristotelia chilensis* and *Quillaja saponaria* in relation to the applied phosphorus concentration (P), chemical root pruning (RP), and their interaction (P × RP) in the distribution of fine, medium, and coarse roots. Significant differences at *p* < 0.05 in bold.

	Source of Variation	Length			Volume		
		Fine	Medium	Coarse	Fine	Medium	Coarse
*A. chilensis*	Phosphorous (P)	0.2792	**0.0021**	0.2954	0.0675	**0.0038**	**0.0004**
	Root Pruning (RP)	0.2472	**0.0380**	0.0807	**0.0485**	**0.0382**	0.9086
	P × RP	0.4802	0.5809	0.4547	0.5076	0.4450	0.8502
*Q. saponaria*	Phosphorous (P)	**0.0003**	**0.0008**	**0.0146**	**0.0001**	**0.0008**	**0.0035**
	Root Pruning (RP)	**0.0322**	**0.0264**	0.1082	**0.0199**	**0.0428**	0.0677
	P × RP	**0.0011**	0.0778	0.0899	0.0516	**0.0379**	0.0343

## Data Availability

The datasets generated for this study are available on request to the corresponding authors.

## Supplementary Materials

The following supporting information can be downloaded at https://www.mdpi.com/article/10.3390/plants14020195/s1: Table S1. Chemical analysis of water used for irrigation during the nursery period; Figure S1: Root pruning effect in *A. chilensis* in relation to P fertilization. A: 0P; B: 15P; C: 60P; D: 120P. For each image, the root on the right represents WoCu and on the left represents WCu; Figure S2: Root pruning effect in *Q. saponaria* in relation to P fertilization. A: 0P; B: 15P; C: 60P; D: 120P. For each image, the root on the right represents WoCu and on the left represents WCu.

## References

[1] Martínez D., Chadwick C., Plaza-Aguilar A. (2023). The Time of Emergence (ToE) of the Andean Mediterranean Sclerophyllous Forest of *Quillaja saponaria* (Mol.) and *Lithraea caustica* (Mol.) Hox. & Arn. For. Ecol. Manag..

[2] Schröter D., Cramer W., Leemans R., Prentice I.C., Araújo M.B., Arnell N.W., Bondeau A., Bugmann H., Carter T.R., Gracia C.A. (2005). Ecosystem Service Supply and Vulnerability to Global Change in Europe. Science.

[3] Rundel P.W., Arroyo M.T.K., Cowling R.M., Keeley J.E., Lamont B.B., Vargas P. (2016). Mediterranean Biomes: Evolution of Their Vegetation, Floras, and Climate. Annu. Rev. Ecol. Evol. Syst..

[4] Miranda A., Syphard A.D., Berdugo M., Carrasco J., Gómez-González S., Ovalle J.F., Delpiano C.A., Vargas S., Squeo F.A., Miranda M.D. (2023). Widespread Synchronous Decline of Mediterranean-Type Forest Driven by Accelerated Aridity. Nat. Plants.

[5] Rojas M., Lambert F., Ramirez-Villegas J., Challinor A.J. (2019). Emergence of Robust Precipitation Changes across Crop Production Areas in the 21st Century. Proc. Natl. Acad. Sci. USA.

[6] Polade S.D., Gershunov A., Cayan D.R., Dettinger M.D., Pierce D.W. (2017). Precipitation in a Warming World: Assessing Projected Hydro-Climate Changes in California and Other Mediterranean Climate Regions. Sci. Rep..

[7] Peñuelas J., Sardans J. (2021). Global Change and Forest Disturbances in the Mediterranean Basin: Breakthroughs, Knowledge Gaps, and Recommendations. Forests.

[8] Hooper D.U., Chapin III F.S., Ewel J.J., Hector A., Inchausti P., Lavorel S., Lawton J.H., Lodge D.M., Loreau M., Naeem S. (2005). Effects of Biodiversity on Ecosystem Functioning: A Consensus of Current Knowledge. Ecol. Monog..

[9] Picard N., Garavaglia V., Ne’eman G., Osem Y. (2021). Mediterranean Forests and the United Nations Sustainable Development Goals. Pines and Their Mixed Forest Ecosystems in the Mediterranean Basin.

[10] Leon-Lobos P., Bustamante-Sanchez M.A., Nelson C.R., Alarcon D., Hasbun R., Way M., Pritchard H.W., Armesto J.J. (2020). Lack of Adequate Seed Supply Is a Major Bottleneck for Effective Ecosystem Restoration in Chile: Friendly Amendment to Bannister et al. (2018). Restor. Ecol..

[11] Acevedo M., Álvarez-Maldini C., Dumroese R.K., Bannister J.R., Cartes E., González M. (2021). Native Plant Production in Chile. Is It Possible to Achieve Restoration Goals by 2035?. Land.

[12] Bannister J., Vargas-Gaete R., Ovalle J., Acevedo M., Fuentes-Ramirez A., Donoso P., Promis A., Smith-Ramirez C. (2018). Major Bottlenecks for the Restoration of Natural Forests in Chile. Restor. Ecol..

[13] Magni C.R., Poch P.L., Espinoza S.E., Yáñez M.A., Martínez E.E., Promis A.A., Mancilla G.A. (2023). Provenance Influences Seed Germination and Phenotypic Responses to Water Restriction in the Endemic *Beilschmiedia miersii* (Gay) Kosterm. Front. For. Glob. Chang..

[14] Montagnoli A., Dumroese R.K., Negri G., Scippa G.S., Chiatante D., Terzaghi M. (2022). Asymmetrical Copper Root Pruning May Improve Root Traits for Reforesting Steep and/or Windy Sites. New For..

[15] Montagnoli A., Dumroese R.K., Terzaghi M., Pinto J.R., Fulgaro N., Scippa G.S., Chiatante D. (2018). Tree Seedling Response to LED Spectra: Implications for Forest Restoration. Plant Biosyst..

[16] Dumroese R.K., Landis T.D., Pinto J.R., Haase D.L., Wilkinson K.W., Davis A.S. (2016). Meeting Forest Restoration Challenges: Using the Target Plant Concept. REFOR.

[17] Cole R.J., Holl K.D., Keene C.L., Zahawi R.A. (2011). Direct Seeding of Late-Successional Trees to Restore Tropical Montane Forest. For. Ecol. Manag..

[18] Landis T.D., Dumroese R.K., Haase D.L. (2010). The Target Plant Concept. Container Tree Nursery Manual, Volume 7: Seedling Processing, Storage, and Outplanting.

[19] Landis T.D., Dumroese R.K., MacLennan L., Fennessy J. (2006). Applying the Target Plant Concept to Nursery Stock Quality. Plant Quality: A Key to Success in Forest Establishment. Proceeding of the COFORD Conference. Tullow, Co Carlow, Ireland, 20–21 September 2005.

[20] Wang F.-X., Wang Z.-Y., Lee J.H.W. (2007). Acceleration of Vegetation Succession on Eroded Land by Reforestation in a Subtropical Zone. Ecol. Eng..

[21] Luoranen J., Rikala R., Konttinen K., Smolander H. (2006). Summer Planting of *Picea abies* Container-Grown Seedlings: Effects of Planting Date on Survival, Height Growth and Root Egress. For. Ecol. Manag..

[22] Hostetler A.N., Morais De Sousa Tinoco S., Sparks E.E. (2024). Root Responses to Abiotic Stress: A Comparative Look at Root System Architecture in Maize and Sorghum. J. Exp. Bot..

[23] Villar-Salvador P., Puértolas J., Cuesta B., Peñuelas J.L., Uscola M., Heredia-Guerrero N., Rey Benayas J.M. (2012). Increase in Size and Nitrogen Concentration Enhances Seedling Survival in Mediterranean Plantations. Insights from an Ecophysiological Conceptual Model of Plant Survival. New For..

[24] Ovalle J.F., Ginocchio R., Arellano E.C., Valenzuela P. (2017). Root Adaptive Management for Improving Plant Quality and Field Performance under Drought: Experiences with Native Tree Species from a South American Mediterranean-Type Ecosystem. Plant Sociol..

[25] Cuesta B., Villar-Salvador P., Puértolas J., Jacobs D.F., Rey Benayas J.M. (2010). Why Do Large, Nitrogen Rich Seedlings Better Resist Stressful Transplanting Conditions? A Physiological Analysis in Two Functionally Contrasting Mediterranean Forest Species. For. Ecol. Manag..

[26] Luis V.C., Puértolas J., Climent J., Peters J., González-Rodríguez Á.M., Morales D., Jiménez M.S. (2009). Nursery Fertilization Enhances Survival and Physiological Status in Canary Island Pine (*Pinus canariensis*) Seedlings Planted in a Semiarid Environment. Eur. J. For. Res..

[27] Herrera-Estrella L., López-Arredondo D. (2016). Phosphorus: The Underrated Element for Feeding the World. Trends Plant Sci..

[28] Landis T.D., van Steenis E. (2004). Macronutrients—Phosphorus. Forest Nursery Notes, Summer 2004.

[29] Wu C., Wei X., Sun H., Wang Z. (2005). Phosphate Availability Alters Lateral Root Anatomy and Root Architecture of *Fraxinus Mandshurica* Rupr. Seedlings. J. Integr. Plant Biol..

[30] Song C.J., Ma K.M., Qu L.Y., Liu Y., Xu X.L., Fu B.J., Zhong J.F. (2010). Interactive Effects of Water, Nitrogen and Phosphorus on the Growth, Biomass Partitioning and Water-Use Efficiency of *Bauhinia faberi* Seedlings. J. Arid Environ..

[31] Trubat R., Cortina J., Vilagrosa A. (2012). Root Architecture and Hydraulic Conductance in Nutrient Deprived *Pistacia lentiscus* L. Seedlings. Oecologia.

[32] Sardans J., Peñuelas J., Rodà F. (2006). Plasticity of Leaf Morphological Traits, Leaf Nutrient Content, and Water Capture in the Mediterranean Evergreen Oak *Quercus ilex* Subsp. Ballota in Response to Fertilization and Changes in Competitive Conditions. Ecoscience.

[33] Oliet J.A., Planelles R., Artero F., Jacobs D.F. (2005). Nursery Fertilization and Tree Shelters Affect Long-Term Field Response of *Acacia salicina* Lindl. Planted in Mediterranean Semiarid Conditions. For. Ecol. Manag..

[34] Baesso B., Chiatante D., Terzaghi M., Zenga D., Nieminen K., Mahonen A.P., Siligato R., Helariutta Y., Scippa G.S., Montagnoli A. (2018). Transcription Factors PRE 3 and WOX 11 Are Involved in the Formation of New Lateral Roots from Secondary Growth Taproot in *A. thaliana*. Plant Biol. J..

[35] Xu D., Miao J., Yumoto E., Yokota T., Asahina M., Watahiki M. (2017). YUCCA9-Mediated Auxin Biosynthesis and Polar Auxin Transport Synergistically Regulate Regeneration of Root Systems Following Root Cutting. Plant Cell Physiol..

[36] Wenny D.L., Liu Y., Dumroese R.K., Osborne H.L. (1988). First Year Field Growth of Chemically Root Pruned Containerized Seedlings. New For..

[37] Ruehle J.L. (1985). The Effect of Cupric Carbonate on Root Morphology of Containerized Mycorrhizal Pine Seedlings. Can. J. For. Res..

[38] Tsakaldimi M.N., Ganatsas P.P. (2006). Effect of Chemical Root Pruning on Stem Growth, Root Morphology and Field Performance of the Mediterranean Pine *Pinus halepensis* Mill. Sci. Hortic..

[39] Aguilera-Rodríguez M., Aldrete A., Vargas-Hernández J.J., López-Upton J., López-López M.Á., Ordaz-Chaparro V.M. (2021). Morphology and Root Growth Potential of *Pinus patula* Produced in Trays with Root Pruning. Agrociencia.

[40] Dumroese R.K., Sung S.-J.S., Pinto J.R., Ross-Davis A., Scott D.A. (2013). Morphology, Gas Exchange, and Chlorophyll Content of Longleaf Pine Seedlings in Response to Rooting Volume, Copper Root Pruning, and Nitrogen Supply in a Container Nursery. New For..

[41] Burdett A.N. (1990). Physiological Processes in Plantation Establishment and the Development of Specifications for Forest Planting Stock. Can. J. For. Res..

[42] Liu J., Bloomberg M., Li G., Liu Y. (2016). Effects of Copper Root Pruning and Radicle Pruning on First-Season Field Growth and Nutrient Status of Chinese Cork Oak Seedlings. New For..

[43] Guerrero P.C., Bustamante R.O. (2009). Abiotic Alterations Caused by Forest Fragmentation Affect Tree Regeneration: A Shade and Drought Tolerance Gradient in the Remnants of Coastal Maulino Forest. Rev. Chil. Hist. Nat..

[44] Fernández M.P., Preller C., Fischer S., Espinoza C., Peña-Rojas K., Menéndez-Miguélez M. (2019). Maqui (*Aristotelia chilensis* [Molina] Stuntz): The Most Antioxidant Wild Berry towards Agricultural Production. Fruits.

[45] Magni C., Espinoza S., Poch P., Abarca B., Grez I., Martínez E., Yáñez M., Santelices R., Cabrera A. (2019). Growth and Biomass Partitioning of Nine Provenances of *Quillaja saponaria* Seedlings to Water Stress. South. For..

[46] Pelah D., Abramovich Z., Markus A., Wiesman Z. (2002). The Use of Commercial Saponin from *Quillaja saponaria* Bark as a Natural Larvicidal Agent against *Aedes aegypti* and *Culex pipiens*. J. Ethnopharmacol..

[47] López M., Abarca B., Espinoza S., Rojas A., Martínez-Herrera E., Yáñez M., Magni C.R. (2024). A Proposed Methodology for the Determination of Seed Sources for Tree Native Species Based on Environmental Variables: The Case of *Quillaja saponaria* Mol. New For..

[48] Razaq M., Zhang P., Shen H.-L., Salahuddin (2017). Influence of Nitrogen and Phosphorous on the Growth and Root Morphology of *Acer mono*. PLoS ONE.

[49] Andivia E., Fernández M., Vázquez-Piqué J. (2011). Autumn Fertilization of *Quercus ilex* ssp. Ballota (Desf.) Samp. Nursery Seedlings: Effects on Morpho-Physiology and Field Performance. Ann. For. Sci..

[50] Constantino V., Motta A.C.V., Barbosa J.Z., Dolinski M.A., Zanette F., Prior S.A. (2018). Initial Growth of *Araucaria angustifolia* Rootstock in Response to Fertilization with Nitrogen, Phosphorus and Potassium. RF.

[51] Grossnickle S. (2005). Importance of Root Growth in Overcoming Planting Stress. New For..

[52] Grossnickle S., Colombo S.J. (2005). Seedling Size and Reforestation Success. How Big Is Big Enough?. The Thin Green Line: A Symposium on the State-of-the-Art in Reforestation, Thunder Bay, ON, Canada, 26–28 Jul 2005.

[53] Qu L., Quoreshi A.M., Koike T. (2003). Root Growth Characteristics, Biomass and Nutrient Dynamics of Seedlings of Two Larch Species Raised under Different Fertilization Regimes. Plant Soil.

[54] Gleeson S.K., Good R.E. (2003). Root Allocation and Multiple Nutrient Limitation in the New Jersey Pinelands. Ecol. Lett..

[55] Singh D.K., Sale P.W.G., Pallaghy C.K., Mckenzie B.M. (2000). Phosphorus Concentrations in the Leaves of Defoliated White Clover Affect Abscisic Acid Formation and Transpiration in Drying Soil. New Phytol..

[56] Marchioretto L.D.R., De Rossi A., Conte E.D. (2020). Chemical Root Pruning Improves Quality and Nutrient Uptake of Cape Gooseberry (*Physalis peruviana*) Seedlings. Sci. Hortic..

[57] Graciano C., Goya J.F., Frangi J.L., Guiamet J.J. (2006). Fertilization with Phosphorus Increases Soil Nitrogen Absorption in Young Plants of *Eucalyptus grandis*. Forest Ecol. Manag..

[58] Seabra C.E.B.C., Osiecka A., Tucci C.A.F., Minogue P.J., Pereira B.F.F., Andersen P.C. (2018). Influence of Phosphorus Limitations on the Growth, Nutrient Partitioning and Physiology of Mahogany (*Swietenia macrophylla* King) Seedlings. J. Plant Nutr..

[59] Trubat R., Cortina J., Vilagrosa A. (2006). Plant Morphology and Root Hydraulics Are Altered by Nutrient Deficiency in *Pistacia lentiscus* (L.). Trees.

[60] Tariq A., Pan K., Olatunji O.A., Graciano C., Li Z., Sun F., Sun X., Song D., Chen W., Zhang A. (2017). Phosphorous Application Improves Drought Tolerance of *Phoebe zhennan*. Front. Plant Sci..

[61] Costa L., Faustino L.I., Graciano C. (2017). The Spatial Distribution of Phosphate in the Root System Modulates N Metabolism and Growth in *Eucalyptus grandis* Young Plants. Trees.

[62] Tariq A., Graciano C., Pan K., Olatunji O.A., Li Z., Sadia S., Zhang Z., Ismoilov K., Ahmed Z., Ullah A. (2022). Phosphorus Fertilization of *Phoebe zhennan* Seedlings under Drought Reduces Nitrogen Assimilation. J. Plant Nutr..

[63] Heydari M.M., Brook R.M., Jones D.L. (2019). The Role of Phosphorus Sources on Root Diameter, Root Length and Root Dry Matter of Barley (*Hordeum vulgare* L.). J. Plant Nutr..

[64] Fernandes A.M., Soratto R.P., Gonsales J.R. (2014). Root Morphology and Phosphorus Uptake by Potato Cultivars Grown under Deficient and Sufficient Phosphorus Supply. Sci. Hortic..

[65] Herdler S., Kreuzer K., Scheu S., Bonkowski M. (2008). Interactions between Arbuscular Mycorrhizal Fungi (*Glomus intraradices*, Glomeromycota) and Amoebae (*Acanthamoeba castellanii*, Protozoa) in the Rhizosphere of Rice (*Oryza sativa*). Soil Biol. Biochem..

[66] Jin J., Wang G., Liu X., Pan X., Herbert S.J. (2005). Phosphorus Application Affects the Soybean Root Response to Water Deficit at the Initial Flowering and Full Pod Stages. Soil Sci. Plant Nutr..

[67] Toro M., Azcon R., Barea J. (1997). Improvement of Arbuscular Mycorrhiza Development by Inoculation of Soil with Phosphate-Solubilizing Rhizobacteria To Improve Rock Phosphate Bioavailability ((Sup32)P) and Nutrient Cycling. Appl. Environ. Microbiol..

[68] Huang B., Nobel P.S. (1992). Hydraulic Conductivity and Anatomy for Lateral Roots of Agave Deserti During Root Growth and Drought-Induced Abscission. J. Exp. Bot..

[69] Fitter A.H., Stickland T.R., Harvey M.L., Wilson G.W. (1991). Architectural Analysis of Plant Root Systems 1. Architectural Correlates of Exploitation Efficiency. New Phytol..

[70] Toca A., Moler E., Nelson A., Jacobs D.F. (2022). Environmental Conditions in the Nursery Regulate Root System Development and Architecture of Forest Tree Seedlings: A Systematic Review. New For..

[71] Kramer-Walter K.R., Bellingham P.J., Millar T.R., Smissen R.D., Richardson S.J., Laughlin D.C. (2016). Root Traits are Multidimensional: Specific Root Length Is Independent from Root Tissue Density and the Plant Economic Spectrum. J. Ecol..

[72] Laliberté E., Lambers H., Burgess T.I., Wright J. (2015). Phosphorus Limitation, Soil-Borne Pathogens and the Coexistence of Plant Species in Hyperdiverse Forests and Shrublands. New Phytol..

[73] Ho M.D., Rosas J.C., Brown K.M., Lynch J.P. (2005). Root Architectural Tradeoffs for Water and Phosphorus Acquisition. Funct. Plant Biol..

[74] Ostonen I., Püttsepp Ü., Biel C., Alberton O., Bakker M.R., Lõhmus K., Majdi H., Metcalfe D., Olsthoorn A.F.M., Pronk A. (2007). Specific Root Length as an Indicator of Environmental Change. Plant Biosyst..

[75] Xu B., Niu F., Duan D., Xu W.-Z., Huang J. (2012). Root Morphological Characteristics of *Lespedeza davurica* (L.) Intercropped with *Bothriochloa ischaemum* (L.) Keng under Water Stress and P Application Conditions. Pak. J. Bot..

[76] Ji L., Attaullah K., Wang J., Yu D., Yang Y., Yang L., Lu Z. (2020). Root Traits Determine Variation in Nonstructural Carbohydrates (NSCs) under Different Drought Intensities and Soil Substrates in Three Temperate Tree Species. Forests.

[77] Padilla F.M., Ortega R., Sánchez J., Pugnaire F.I. (2009). Rethinking Species Selection for Restoration of Arid Shrublands. Basic Appl. Ecol..

[78] Wright I.J., Westoby M. (1999). Differences in Seedling Growth Behaviour among Species: Trait Correlations across Species, and Trait Shifts along Nutrient Compared to Rainfall Gradients. J. Ecol..

[79] Birouste M., Zamora-Ledezma E., Bossard C., Pérez-Ramos I.M., Roumet C. (2014). Measurement of Fine Root Tissue Density: A Comparison of Three Methods Reveals the Potential of Root Dry Matter Content. Plant Soil.

[80] Rewald B., Rechenmacher A., Godbold D.L. (2014). It’s Complicated: Intraroot System Variability of Respiration and Morphological Traits in Four Deciduous Tree Species. Plant Physiol..

[81] Olmo M., Lopez-Iglesias B., Villar R. (2014). Drought Changes the Structure and Elemental Composition of Very Fine Roots in Seedlings of Ten Woody Tree Species. Implications for a Drier Climate. Plant Soil.

[82] King J.S., Albaugh T.J., Allen H.L., Buford M., Strain B.R., Dougherty P. (2002). Below-Ground Carbon Input to Soil Is Controlled by Nutrient Availability and Fine Root Dynamics in Loblolly Pine. New Phytol..

[83] Brunner I., Herzog C., Dawes M.A., Arend M., Sperisen C. (2015). How Tree Roots Respond to Drought. Front. Plant Sci..

[84] Acevedo M., Álvarez C., Cartes E., Dumroese R.K., González M. (2020). Production and Establishment Techniques for the Restoration of *Nothofagus alessandrii*, an Endangered Keystone Species in a Mediterranean Forest. New For..

[85] González M., Ríos D., Peña Rojas K., García E., Acevedo M., Cartes E., Sánchez Olate M. (2020). Efecto de la concentración de fósforo y calcio sobre atributos morfo-fisiológicos y potencial de crecimiento radical en plantas de *Aextoxicon punctatum* producidas a raíz cubierta en la etapa de endurecimiento. Bosque.

[86] Dumroese R.K., Montville M.E., Pinto J.R. (2015). Using Container Weights to Determine Irrigation Needs: A Simple Method. Native Plants J..

[87] Alvarez-Maldini C., Acevedo M., Dumroese R.K., González M., Cartes E. (2020). Intraspecific Variation in Drought Response of Three Populations of *Cryptocarya alba* and *Persea lingue*, Two Native Species from Mediterranean Central Chile. Front. Plant Sci..

[88] Galmés J., Cifre J., Medrano H., Flexas J. (2005). Modulation of Relative Growth Rate and Its Components by Water Stress in Mediterranean Species with Different Growth Forms. Oecologia.

[89] Bergerud W.A. (1996). Introduction to Logistic Regression Models with Worked Forestry Examples: Biometrics Information Handbook No.7.

