# Supplementary material for: Phosphorus Fertilization and Chemical Root Pruning: Effects on Root Traits During the Nursery Stage in Two Mediterranean Species from Central Chile
Source: Plants (Basel). 2025 Jan 12;14(2):195. doi: 10.3390/plants14020195 (PMC11768390; doi:10.3390/plants14020195)
Supplement: Supplementary file 1 [file plants-14-00195-s001.zip › plants-3387240-supplementary.pdf]

Table S1. Chemical analysis of water used for irrigation during the nursery period.

**Parameter**	**Concentration (mg L^{-1})**
pH	7.09
CE	0.03
NH_4^+	0.01
NO_3^-	12.00
P	0.01
K	3.60
Ca	19.70
Mg	9.67
SO_4	9.80
B	0.04

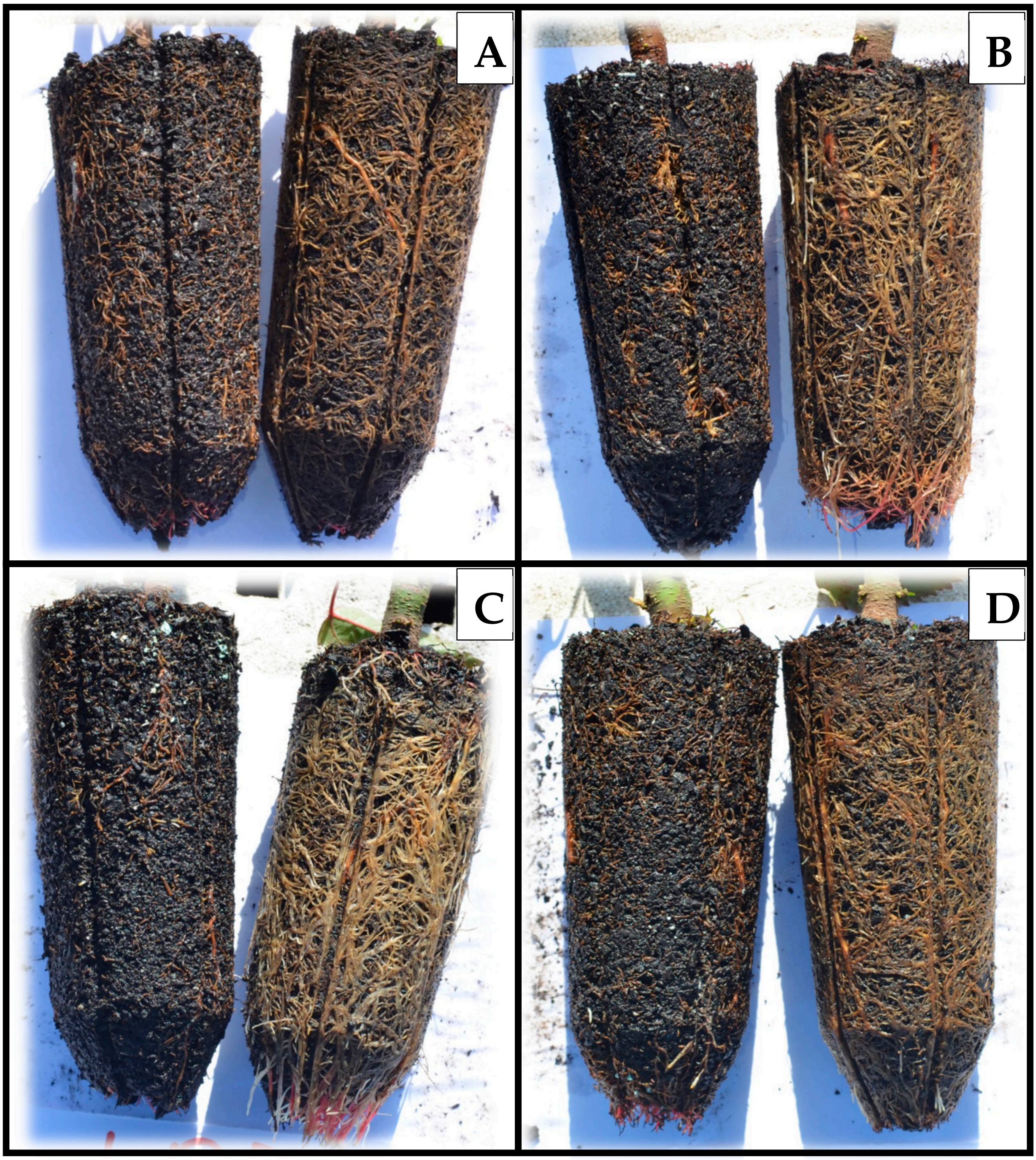

Figure S1. Root pruning effect in *A. chilensis* in relation to P fertilization. A: 0P; B: 15P; C: 60P; D: 120P. For each image, the root on the right represents WoCu and on the left represents WCu.

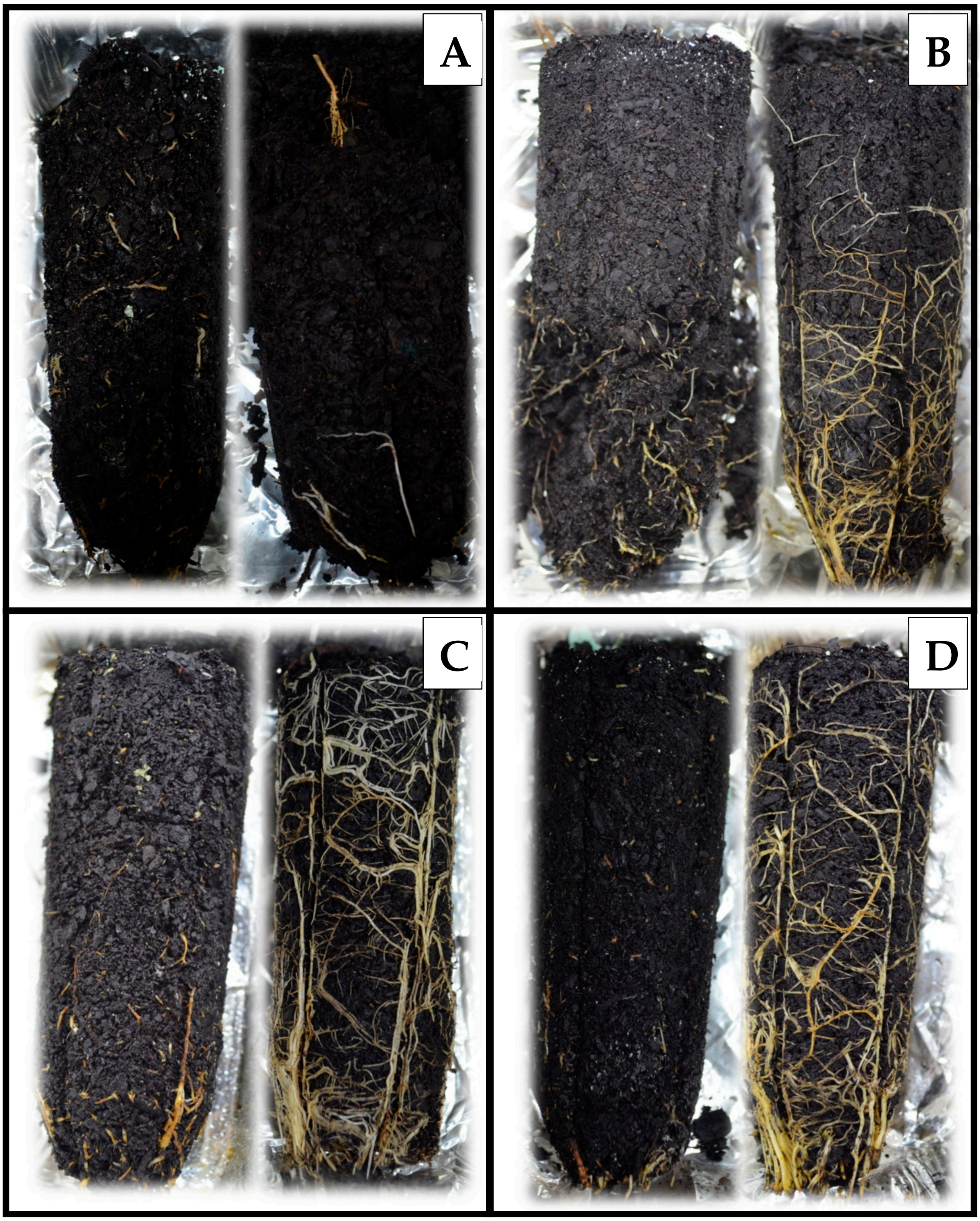

Figure S2. Root pruning effect in *Q. saponaria* in relation to P fertilization. A: 0P; B: 15P; C: 60P; D: 120P. For each image, the root on the right represents WoCu and on the left represents WCu.
